# Punicalagin Targets Atherosclerosis: Gene Expression Profiling of THP-1 Macrophages Treated with Punicalagin and Molecular Docking

**DOI:** 10.3390/cimb44050145

**Published:** 2022-05-12

**Authors:** Etimad Huwait, Sanaa Almowallad, Rehab Al-Massabi, Salma Saddeek, Kalamegam Gauthaman, Alexandre Prola

**Affiliations:** 1 Department of Biochemistry, Faculty of Sciences, King Abdul Aziz University, Jeddah 22254, Saudi Arabia; 2 Cell Culture Unit & Experimental Biochemistry Unit, King Fahad Medical Research Centre, King Abdul Aziz University, Jeddah 22254, Saudi Arabia; 3 Department of Biochemistry, Faculty of Sciences, University of Tabuk, Tabuk 47512, Saudi Arabia; rf-saif@ut.edu.sa; 4 Department of Chemistry, Faculty of Sciences, University of Hafr Al Batin, Hafr Al Batin 39524, Saudi Arabia; salmayms@uhb.edu.sa; 5 Department of Pharmacology, Saveetha Dental College and Hospital, Saveetha Institute of Medical and Technical Sciences, Chennai 600077, India; gauthamank.sdc@saveetha.com; 6 Pharmaceutical Division, Nibblen Life Sciences Private Limited, Chennai 600061, India; 7 RMD Specialties Hospital, RMD Academy for Health (A Unit of Pain and Palliative Care Trust), Chennai 600017, India; 8 Department of Cell Physiology and Metabolism, Faculty of Medicine, University of Geneva, 1 rue Michel-Servet, CH-1211 Geneva, Switzerland; alexandre.prola@unige.ch

**Keywords:** atherosclerosis, THP-1 macrophages, punicalagin, gene expression, molecular docking

## Abstract

Atherosclerosis is an important cause of cardiovascular disorders worldwide. Natural botanical drugs have attracted attention due to their antioxidant, anti-inflammatory, and antiatherogenic properties in the treatment of atherosclerosis. Punicalagin is the major bioactive component of pomegranate peel, and has been shown to have antioxidant, anti-inflammatory, antiviral, anti proliferation, and anticancer properties. To explore its antiatherogenic properties at a molecular level, we investigated the genome-wide expression changes that occur in differentiated THP1 cells following treatment with a non-toxic dose of punicalagin. We also conducted a molecular docking simulation study to identify the molecular targets of punicalagin.

## 1. Introduction

Cardiovascular disease (CVD) is the leading cause of death worldwide [1,2]. One of the most important causes of CVD is atherosclerosis. Atherosclerosis is a progressive, chronic inflammatory condition characterized by the initiation and perpetuation of atherosclerotic lesions, which may erode or rupture leading to clinical events such as angina, myocardial infarction, or cerebrovascular attack [3]. It is initiated through the activation of the arterial endothelium by several risk factors leading to the recruitment of immune cells–particularly T-lymphocytes and monocytes. The latter differentiate into macrophages–a process that is accompanied by increased expression of scavenger receptors–and then transform into lipid-loaded foam cells [4]. Immune competent cells producing proinflammatory cytokines are abundant in atherosclerotic lesions and are proposed to play an important role in the progression of the disease [4,5].

Polyphenols are gaining increasing acceptance as therapeutic agents for use in diverse diseases, including CVD [6,7]. Polyphenols naturally exist in plants and plant products, including fruits, vegetables, nuts, herbs, cocoa, and tea. Historically, biologic actions of polyphenols have been attributed to antioxidant activities, but recent evidence suggests that immunomodulatory and vasodilatory properties of polyphenols may also contribute to CVD risk reduction [8]. Ellagitannins are a family of bioactive polyphenols found in fruits and nuts such as pomegranates, black raspberries, raspberries, strawberries, walnuts, and almonds [9]. Ellagitannins are hydrolysable tannins that release ellagic acid on hydrolysis. Pomegranate (*P. granatum* L.) juice, obtained by squeezing the whole fruit, has the highest concentration of ellagitannins than any commonly consumed juice, and contains the unique ellagitannin, punicalagin. Among the pomegranate ellagitannins, punicalagin is the largest polyphenol, and is reported to be responsible for more than half the potent antioxidant activity of the juice [10].

Several studies support the preventive and therapeutic effects of both pomegranates and punicalagin against atherosclerosis [11]. Pomegranate juice (PJ) supplementation has been shown to have potent antiatherogenic effects in healthy humans and in atherosclerotic mice, which may be attributable to its antioxidative properties [12]. PJ supplementation reduced atherosclerotic lesions by 44% and the number of foam cells were reduced in the lesions in atherosclerotic apoE-deficient mice compared to the control group. In addition, oral administration of PJ to hypercholesterolemic, LDL-receptor-deficient mice at various stages of the disease significantly reduced the progression of atherosclerosis [13].

Punicalagin is known for its hypoglycemic, cardioprotective, and antiatherogenic activities [14,15]. Punicalagin-rich pomegranate fruit extract has been shown to reverse the proatherogenic effects associated with perturbed flow in cellular models [16], to protect macrophage cells from lipid accumulation and foam cell formation, and to reduce the development of atherosclerosis [17]. Specifically, punicalagin binds to apolipoprotein B-100 and induces LDL influx into macrophages to a level that prevents their transformation into foam cells [18].

Punicalagin-rich extracts can retard the development of vascular dysfunction and atherosclerosis in early stages in subjects eating a fat-rich diet [12]. Punicalagin has been shown to attenuate LPS-induced inflammatory responses in RAW264.7 macrophages [13]. Supplementation with punicalagin and hydroxytyrosol exerted anti-atherosclerotic effects by improving endothelial function, blood pressure, and levels of circulating oxidized low-density lipoproteins (oxLDL)—an important marker of atherogenesis [14].

The antiatherogenic effect of punicalagin is believed to primarily stem from its antioxidant capacity [14]. However, antioxidant activity cannot be the sole explanation for punicalagin’s cellular effects, since it is poorly absorbed through the gut into the bloodstream and extensively metabolized in the small intestine, liver, and colon; thus, its bioavailability is often poor [19]. Recent studies suggest that the cellular effects of punicalagin can also be mediated by its interaction with specific proteins, or by its ability to modulate the expression of certain inflammatory mediators [18,20,21]. To begin filling the gaps that exist in understanding the mechanism of action of punicalagin as an antiatherogenic compound at the molecular level, we decided to combine molecular docking with microarray analysis on THP-1 macrophages.

## 2. Materials and Methods

### 2.1. Chemicals

Phorbol 12-myristate 13-acetate (PMA) (Cat No. P8139) and punicalagin (≥98% HPLC, Cat No. P0023) were obtained from Sigma-Aldrich, Saint Louis, MO, USA. Dimethyl sulfoxide (DMSO, D12345) was obtained from Invitrogen. A 10 μM main stock of punicalagin was prepared by diluting 10 milligrams of punicalagin with 921 μL of DMSO.

### 2.2. THP1 Cell Culture

THP-1 authenticated cell lines (RRID: CVCL_0006) (Homo sapiens monocytes) were a generous gift from Molecular Biomedicine Unit, King Faisal Specialist Hospital & Research Centre (KFSHRC), Riyadh, KSA. Cells were cultured in RPMI 1640 supplemented with 10% heat-inactivated FCS, 2 mmol/L glutamine, and 100 μg/mL penicillin-streptomycin. Cells were maintained in a humidified incubator at 37 °C, 5% (*v*/*v*) CO_2_. The cells were sub-cultured when they reached approximately 80% confluence (8 × 10^5^ cells/mL). To promote differentiation to macrophage, confluent THP-1 cells (5–8 × 10^5^ cells/mL) were treated with 160 nM PMA for 24 h [22]. An in vivo mice study had used 140 μg/100 μL (1.4 μg/μL) of punicalagin as subcutaneous dose [12]. We chose the 10 μM concentration of punicalagin based on earlier published [23] in vitro work and from our preliminary dose range determination studies, as well as by extrapolations of the in vivo study. Therefore, THP-1 cells were treated with 10 μM punicalagin for 24 h and control cells were treated with vehicle (Dimethyl sulfoxide), and their levels were maintained at non-toxic levels (0.025–0.05%) to the cells.

### 2.3. Total Cellular RNA Preparation from the Control and Treated THP1 Cells

Total RNA was isolated from ~2 × 10^6^ treated (punicalagin) and control (DMSO) THP-1 cells after harvesting them by scraping. Total RNA was extracted using a Qiagen RNeasy Mini Kit (Qiagen, Hilden, Germany), according to the manufacturer’s instructions. RNA concentrations were determined using a NanoDrop ND-1000 spectrophotometer (NanoDrop Technologies, Wilmington, DE, USA).

### 2.4. Preparation of the Sense Strand DNA for Microarray Gene Expression Analysis

Transcriptional expression profiling was performed with Affymetrix GeneChips (Gene 1.0ST, Affymetrix, Santa Clara, CA, USA) according to the conventional Affymetrix eukaryotic RNA labelling protocol (Affymetrix). Briefly, 250 ng of total RNA isolated from the control (DMSO) and treated (punicalagin) THP1 cells was first converted to single-stranded sense strand DNA (cDNA) in two cycles using the whole transcript (WT) cDNA synthesis amplification kit and sample clean-up module. In the first cycle, the total RNA was converted to double-stranded cDNA using random hexamers tagged with a T7 promoter sequence. Each strand of the double-stranded cDNA was then used as a template to synthesize antisense RNA (cRNA). In the second cycle, the cRNA was reverse-transcribed into sense strand DNA in the presence of random hexamers (3 g/mL) and dNTPs mix (10 mM).

### 2.5. Sense Strand DNA Labelling and Hybridization Gene 1.0 S.T Arrays

The sense strand DNA was cleaned up using the sample clean-up kit and then cleaved into small fragments using a mixture of UDP and apurinic/apyrimidinic endonuclease 1. The fragmented sense strand DNA was then end-labelled through a terminal transferase reaction that incorporates biotinylated di-deoxynucleotides using the WT terminal labelling kit. The fragmented biotinylated sense strand DNA was then hybridized to the Affymetrix Human Gene 1.0S.T array at 45 °C for 16 h in a hybridization Oven 640. After hybridization, the arrays were stained and then washed in the Affymetrix Fluidics Station 450 under standard conditions. The stained arrays were then scanned at 532 nm using an Affymetrix GeneChip Scanner 3000, and CEL files for each array were generated using the Affymetrix Gene-Chip^®^ Operating Software (GCOS). GCOS defines the probe cells and computes an intensity for each cell; complete probe array images were saved with a data image file extension (.dat, cel).

### 2.6. Microarray Data Normalization and Analysis

Affymetrix CEL files were used for raw data for image analysis and probe quantification using Partek Genome Suit 7.0. Normalization was done with the Robust Multi-chip Average (RMA) program that processes a group of CEL files simultaneously. The default options of background correction, quantile normalization, and log transformation were used. Calculated raw probe intensity data was used to derive fold change and *p*-value. Principal component analysis (PCA) was performed on all probes to visualize high-dimensional data and assess quality control, as well as overall variance in gene expression between the two treatments. Analysis of variance (ANOVA) was applied on the complete data set and the list of differentially expressed genes (DEGs) was then generated using a false-discovery rate (FDR) of 0.05 with 2-fold change cut-off. Unsupervised two-dimensional average linkage hierarchical clustering was performed using Spearman’s correlation as a similarity matrix.

### 2.7. Molecular Pathway Analysis

Ingenuity Pathways Analysis (IPA) software version 338830M (Ingenuity Systems, Redwood City, CA, USA) was used to find significant canonical pathways, biological networks, biological functions, and phenotypes/disease associated with present study. DEGs along with their corresponding Affymetrix probe set ID/gene symbol/Entrez gene ID as clone identifiers, *p*-values, and fold change values were uploaded into IPA for functional analysis. The percentage and number of uploaded genes/molecules matching to genes of a canonical pathway were measured as Z-score, ratio, or Fisher’s exact test for significance. The Molecule Activity Predictor was employed to predict the activation or suppression effects of a gene/molecule on other molecules of pathway.

### 2.8. Real-Time PCR Validation

Validation of the microarray data using quantitative real-time PCR (qRT–PCR) was carried out in StepOnePlusTM RT-PCR System (ThermoFisher, Waltham, MA, USA). Total RNA isolated and quality checked for the microarray experiments was used for cDNA synthesis for the qRT–PCR experiments. Manufacturer’s protocol was followed for cDNA synthesis using ImProm-II™ Reverse Transcription System kit (Promega, Madison, WI, USA). The primer pairs listed in Table 1 were used for amplification of the appropriate target and endogenous control genes. All experiments were done in triplicates biological samples, data analyses were performed by Microsoft Excel 2010, and data were plotted by GraphPad Prism 8 (GraphPad Software, Inc., San Diego, CA, USA).

### 2.9. LXR/RXR Activation and Network Interaction

The LXR/RXR activation pathway in macrophage was generated using Ingenuity Target Explorer (QIAGEN, Inc., https://targetexplorer.ingenuity.com/ (accessed on 12 July 2021)). Network interaction between the 15 most differently expressed genes in (LXR/RXR) activation pathway was evaluated using GeneMANIA web tool (https://genemania.org/ (accessed on 16 July 2021)).

### 2.10. Molecular Docking

The X-ray crystal structure of all receptors selected for the study were downloaded from Protein Data Bank. Homology modeled structure of MSR1 macrophage scavenger receptor 1- 3D structure was modeled based on homology using SWISS-MODEL. The three-dimension structures of CD36, TLR4, MSR1, LRP1, NR1H3, PPARγ, and TRAF1 were defined as target macromolecules in Autodock Vina Wizard of (PyRx, RRID: SCR_018548) software. The three-dimensional structure of compounds (palmitic acid, GW3965, pinocembrin, lobeglitazone and punicalagin) were optimized in Discovery Studio Visualizer software (version 2.5) and defined as the ligands for docking using PyRx. The active site analysis was conducted by calculating (x, y, and z coordinates) of palmitic acid, GW3965, pinocembrin, and lobeglitazone in the PyRx software for CD36, NR1H3, TLR4, and PPARγ, respectively. Entire receptors surfaces were used for identifying the active site in the case of MSR1, LRP1, and TRAF1. Blind docking was performed for the receptors MSR1, LRP1, and TRAF1 as no ligands were known. Coordinates of (centre_x, y, z) and dimensions in A (x, y, z) were maximized to cover the entire receptor structure to identify the active site/docking sites. Energy minimization was performed using the “uff” forcefield and conjugate gradients optimization algorithm for standard and test compounds before docking.

## 3. Results

### 3.1. Normalization and Visualization

**Figure 1:**
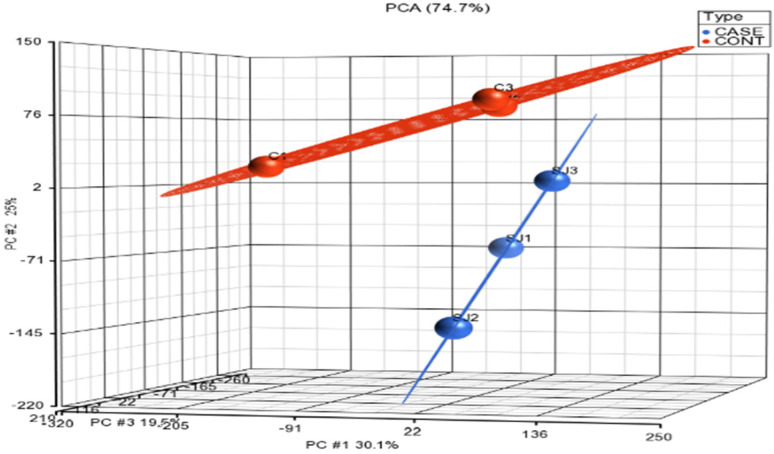
PCA, a 3D scatter plot showing similarity of expression profiles of samples. Punicalagin treated samples are indicated by blue dots and control samples are indicated by red dots.

Microarray data were initially visualized using a principal components analysis scatter (PCA) plot (Figure 1). The data from the control group (n = 3) are shown in red, whereas those in the treated group (n = 3) are indicated in blue. Each ball represents gene expression data generated from a sample that was applied on a gene chip. The results of PCA of transcriptomic data as per their overall expression pattern showed that the samples from the same type of treatment cluster together. From the plot, sample outliers were not detected both in the control and in treated group. The control group is also distinguishably separated from those of the treated samples.

**Figure 2:**
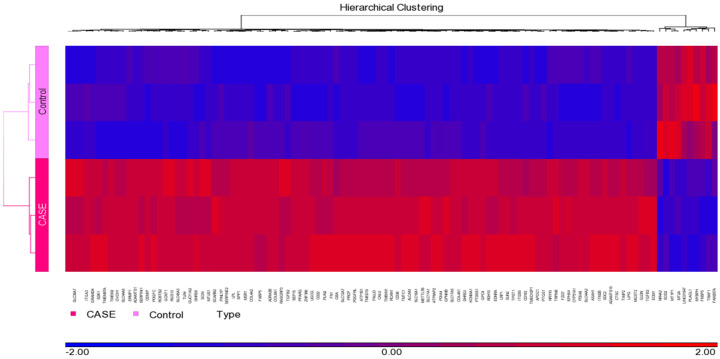
Hierarchical clustering and functional analysis of selected genes significantly differentially expressed in treated cell lines using Affymetrix Human ST 1.0 array and Partek GS 7.0 software.

Figure 2 shows hierarchical clustering of differentially expressed genes in THP1 macrophages in response to treatment with punicalagin. Each lane represents an array. Up-regulated genes in treated samples are shown in red, and down-regulated genes are shown in purple. The pattern of gene expression was similar in the biological replicates. Comparison of the genome-wide expression of treated and control groups by ANOVA revealed 373 differentially expressed genes, including 347 up-regulated and 26 down-regulated with a cut-off of *p* value < 0.05 and fold change more than ±2 (Supplementary Table S1). The top 20 up- and down-regulated genes are represented in Table 2 and Table 3, respectively. FABP4, CD36, MSR1, LPL, and TDO2 were upregulated more than ten-fold in the treated population. Genes that were significantly downregulated include metallothionein genes MT2A and MT1P1.

### 3.2. Identification of Canonical Pathways and Associated Disease Functions

**Figure 3:**
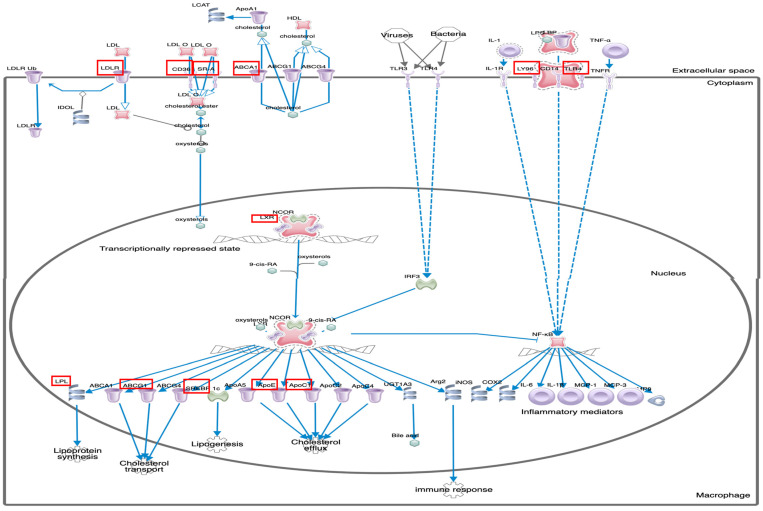
LXR/RXR Activation Pathway in macrophage. Showing predicted activation effect of detected 15 molecules framed in red (ABCG1, AGT, APOC1, APOE, CD36, CYP51A1, HMGCR, LDLR, LPL, LY96, MSR1, NR1H3(LXR), SCD, SERPINF1, TLR4) with ratio: 0.124, z-score: 1.26 and −log (*p*-value): 8.97.

**Figure 4:**
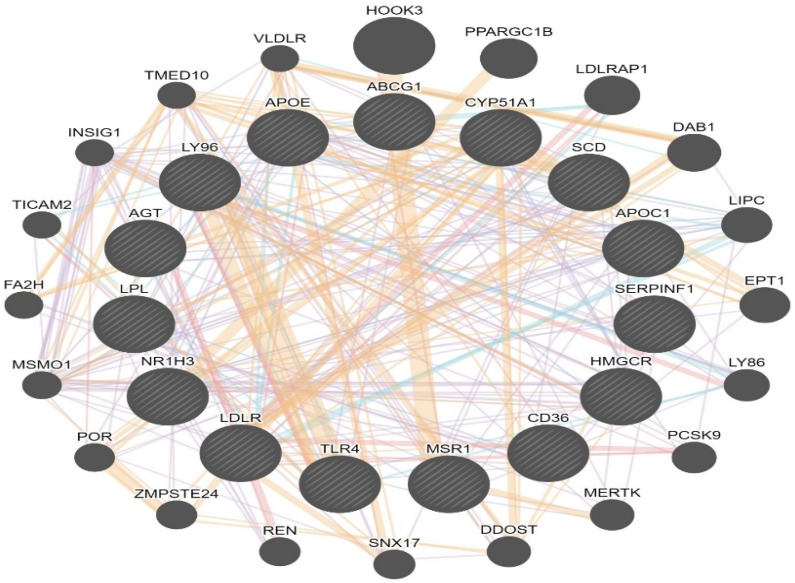
Network of differently expressed genes in the (LXR/RXR) activation pathway. Genes with ratio: 0.615, z-score: 2.8 and −log (*p*-value): 11.3 (ABCG1, AGT, APOC1, APOE, CD36, CYP51A1, HMGCR, LDLR, LPL, LY96, MSR1, NR1H3, SCD, SERPINF1, TLR4), showing 20 related genes and 301 total links. ABCG1: ATP binding cassette subfamily G member 1, CYP51A1: cytochrome P450 family 51 subfamily A member 1, SCD: stearoyl-CoA desaturase, APOC1: apolipoprotein C1, SERPINF1: serpin family F member, HMGCR: 3-hydroxy-3-methylglutaryl-CoA reductase, CD36: CD36 molecule, MSR1: macrophage scavenger receptor 1, TLR4: toll like receptor 4, LDLR: low density lipoprotein receptor, NR1H3: nuclear receptor subfamily 1 group H member 3, LPL: lipoprotein lipase, AGT angiotensinogen, LY96 lymphocyte antigen 96, APOE apolipoprotein E, HOOK3: hook microtubule-tethering protein 3, PPARGC1B: PPARG coactivator 1 beta, LDLRAP1: low density lipoprotein receptor adaptor protein 1, DAB1: DAB1, reelin adaptor protein, LIPC: lipase C, hepatic type, EPT1: ethanolaminephosphotransferase 1, LY86: lymphocyte antigen 86, PCSK9: proprotein convertase subtilisin/kexin type 9, MERTK: MER proto-oncogene, tyrosine kinase, DDOST: dolichyl-diphosphooligosaccharide--protein glycosyltransferase noncatalytic subunit, SNX17: sorting nexin 17, REN renin, ZMPSTE24: zinc metallopeptidase STE24, POR: cytochrome p450 oxidoreductase, MSMO1: methylsterol monooxygenase 1, FA2H: fatty acid 2-hydroxylase, TICAM2: toll like receptor adaptor molecule 2, INSIG1: insulin induced gene 1, TMED10: transmembrane p24 trafficking protein 10, VLDLR: very low density lipoprotein receptor. Genes uses for research are indicated with stripes. Line color indicates the nature of the identified interaction: purple lines: co-expression; orange line: predicted; cyan: pathway; pink: physical interactions; and blue: co-localization.

Ingenuity pathway analysis (IPA) on the 373 upregulated genes was carried out to elucidate the affected biological processes. This analysis revealed canonical pathways such as Cholesterol Biosynthesis I, II, III, super pathway of cholesterol biosynthesis, and LXR/RXR activation, (Table 4, Figure 3) (Supplementary Table S2). Network interaction between the 15 most differently expressed genes in (LXR/RXR) the activation pathway is represented in (Figure 4), showing 20 related genes and 301 total links related to the LXR/RXR pathway. IPA also revealed cell movement, migration of cells, leukocyte migration, non-hematological solid tumor, non-hematologic malignant neoplasm, development of vasculature, cancer, solid tumor, malignant solid tumor, synthesis of lipid, etc., as most associated diseases or functions (Supplementary Tables S3 and S4).

### 3.3. Validation of Microarray Data

**Figure 5:**
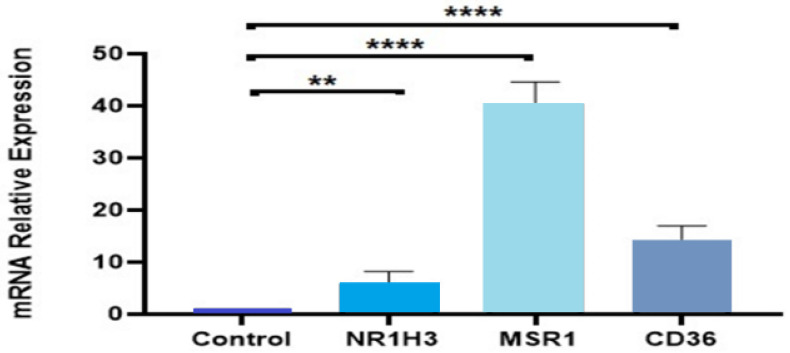
Validation of the microarray data using quantitative real-time PCR. The bar chart shows the gene expression patterns (presented as fold change) of selected significantly up-regulated genes NR1H3 (LXR), MSR1 (SR-A), and CD36 calculated using quantitative real-time PCR. All PCR data were normalized to the intensity of GAPDH as a housekeeping gene. Data represented as mean + SEM following three independent experiments. Statistical analysis was performed using a one-way ANOVA with a Sidak’s test analysis where **: *p* < 0.01, ****: *p* < 0.0001.

The expression of selected up-regulated genes NR1H3 (LXR), MSR1 (SRA), and CD36 relative to that of GADPH was also quantitated using real-time RT–PCR to validate findings from the microarray data. As shown in Figure 5, a robust increase in mRNA derived from these three genes was observed in the punicalagin treated population vs. control. CD36 showed approximately a 13-fold increase in transcript quantities, whereas MSR1 and NR1H3 mRNA increased by 140 folds and 5 folds, respectively, in the treated THP1 macrophage population as compared to the control cells, thus corroborating the increase in mRNA levels of these genes observed following microarray analysis.

### 3.4. Molecular Docking Analysis

**Figure 6:**
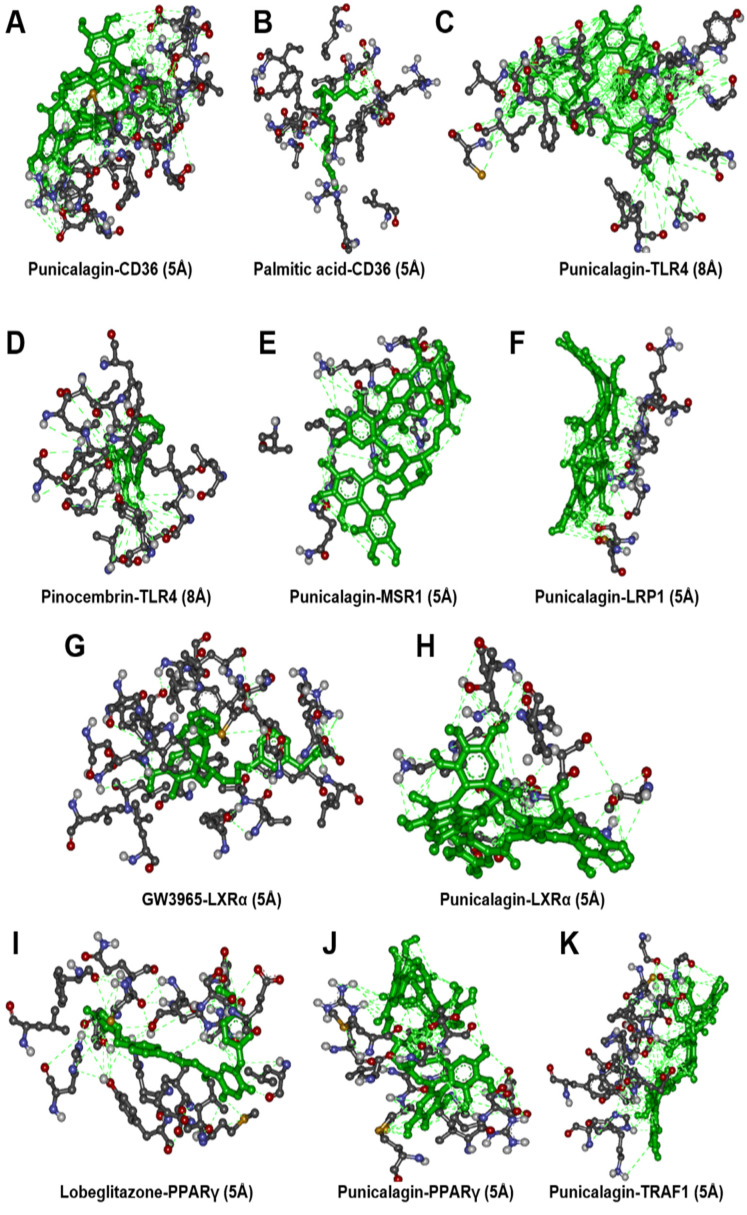
Molecular docking analysis of punicalagin. Molecular docking analysis of punicalagin and respective ligands (represented in green) to CD36 (**A**,**B**), TLR4 (**C**,**D**), MSR1 (**E**), LRP1 (**F**), NR1H3 (**G**,**H**), PPARγ (**I**,**J**), and TRAF1 (**K**).

To identify the molecular target of punicalagin, we conducted molecular docking simulation study. According to the microarray analysis, we focused on seven key receptors: CD36 (Cluster of Differentiation 36), TLR4 (Toll-Like Receptor 4), MSR1 (Macrophage Scavenger Receptor 1), LRP1 (LDL Receptor Related Protein 1), NR1H3 (Nuclear Receptor Subfamily 1 Group H Member 3), PPARγ (Peroxisome Proliferator-Activated Receptor gamma), and TRAF1 (TNF receptor-associated factor 1) (Table 5). We found a significant binding affinity of punicalagin with CD36 (−9.3 Kcal/mol) by establishing hydrogen bond interactions. Binding of CD36 was computed with its well-described ligand palmitic acid, and observed lower binding affinity compared to punicalagin (−6.8 Kcal/mol). Interestingly, it was found that these two CD36-binding molecules exhibit distinct interaction profiles with different binding sites (Figure 6A,B).

In the case of TLR4, pinocembrin–an antagonist for TLR4–showed binding affinity as (−8.2 Kcal/mole), whereas punicalagin showed a slightly higher value as (−9.0 Kcal/mole). Interaction profiling of pinocembrin and punicalagin showed a maximum of seven common hydrogen bonding interactions (Figure 6C,D). This observation suggests that punicalagin is a high affinity inhibitor of TLR4 receptors. As MSR1 structure is not experimentally solved by X-ray crystallography and nuclear magnetic resonance technique, the computational technique of homology modelling was used for 3D structure determination of MSR1. Punicalagin binding affinity was observed to be (−7.4 Kcal/mol). It was observed that punicalagin directly interacted with hydrogen bonding to amino acid residues (Figure 6E).

The binding affinity of punicalagin to LRP1 was estimated to be (−6.4 Kcal/mole), involving hydrogen bonding interactions with amino acid residues in LRP1 (Figure 6F). The binding affinity of punicalagin to the NR1H3 receptor was (−7.1 Kcalcal/mole). In comparison, the binding affinity of the NR1H3 agonist GW3965 was (−13.9 kcal/mole). Interestingly, there was not a single common interaction observed between these two molecules (Figure 6G,H), suggesting that punicalagin could interact with NR1H3 in a different way than other NR1H3 agonists.

In the case of PPARγ, lobeglitazone–an agonist for PPARγ–demonstrated binding affinity of (−8.6 Kcal/mole), whereas punicalagin had a slightly higher value of (−8.7 Kcal/mole). Interaction profiling indicated a maximum of three common hydrogen bonding interactions between lobeglitazone and punicalagin (Figure 6I,J). This indicated that punicalagin might be acting as an agonist in the same way as a lobeglitazone against PPARγ. On the other hand, punicalagin binding affinity against TRAF1 was observed to be (−9.0 Kcal/mol). Its interaction profiling showed hydrogen bonding interaction as in (Figure 6K).

## 4. Discussion

Macrophages are central to the initiation and progression of atherosclerosis and can be highly appropriate targets for antiatherogenic therapy. Punicalagin was demonstrated to exert promising anti-atherosclerotic effects on macrophages, but molecular targets remain to be identified. In this study, we performed the first large-scale gene expression analysis to gain insight into the mechanism of action of punicalagin. Key regulators of identified pathways were then subjected to in silico docking analysis to predict the molecular target of punicalagin. Our results showed that punicalagin anti-atherosclerotic properties rely on multiple targets in THP-1 macrophages. Indeed, we predicted that punicalagin regulates the activity of CD36, TLR4, MSR1, LRP1, NR1H3, PPARγ, and TRAF1. These predictions were strongly supported by transcriptomic analyses showing that punicalagin upregulates the expression of genes involved in cholesterol efflux and lipid metabolism, and reduces the expression of genes involved in inflammation, proliferation, cell migration, and adhesion.

The results of the transcriptomic analyses showed that among the top 20 significantly upregulated genes in response to punicalagin, several were associated with cholesterol or lipids metabolism (FABP4, CD36, LPL, FADS2). This was further supported by ingenuity pathway analysis that revealed that the four most modified canonical pathways in response to punicalagin treatment were associated to cholesterol synthesis (cholesterol biosynthesis pathway I, II, and III and super pathway of cholesterol biosynthesis) and that lipid synthesis is one of the top modified functions. These results are in line with previous studies that revealed a reduction of circulating cholesterol levels following punicalagin treatment, resulting from a reduction of cholesterol synthesis and accumulation in macrophage, associated with an increase of LDL influx to macrophages [12,15,23]. Molecular docking analysis revealed four high-probability targets of punicalagin involved in cholesterol and lipid metabolism: CD36, LRP1, NR1H3, PPARγ. The interaction of punicalagin with CD36 is of particular interest, as the binding is predicted to be higher than with palmitate–the traditional ligand of CD36 [27]. Future studies are warranted to support this finding.

Transcriptomic analyses also support previous observations showing a reduction of proliferation, migration, and adhesion in cancer cells [22,28,29] and macrophages [24] in response to punicalagin treatment. Indeed, among the top 20 significantly upregulated genes in response to punicalagin, several were associated with cell proliferation and migration (CEMIP, GPC4, FCER2, TDO2, MTUS1, PDGFRL, MMP1) or adhesion (MSR1). This was further supported by ingenuity pathway analysis that revealed that some modified canonical pathway in response to punicalagin treatment were associated to proliferation, migration, and adhesion (Granulocyte Adhesion and Diapedesis, Colorectal Cancer Metastasis signalling, agranulocyte Adhesion and Diapedesis Leukocyte Extravasation Signalling). Importantly, cell movement and migration of cells were among the top modified functions. Molecular docking analysis predicted a high likelihood for punicalagin binding to MSR1. This could represent a key target for punicalagin, as this receptor has been showed to regulate the PI3K/AKT/GSK3β/β-catenin pathway, which is involved in lipid metabolism, proliferation, adhesion, and inflammation [22,30,31,32].

Finally, our transcriptomic analyses also confirm the anti-inflammatory role of punicalagin [18,20,33], as evidenced by the fact that among the top 20 significantly downregulated genes, 10 were associated with inflammation (TRAF1, FKBP5, MT2A, MT1P1, PLAGL1, NR4A2, BCL3, MT1X, PIM2, and HSPA4L). Molecular docking analyses predicted that TLR4 and TRAF1, two important regulators of inflammation [34,35], could be targeted by punicalagin. Further studies are needed to experimentally confirm these results. Interestingly, while the antiatherogenic effect of punicalagin was suggested to rely on its antioxidant capacity [14], transcriptomic analyses did not reveal important modifications of genes involved in the antioxidant response. This result supports previous molecular docking analyses that revealed no significant binding of punicalagin to key antioxidant enzymes [36], and reinforces the importance of punicalagin’s effect on lipid metabolism and cell inflammation, proliferation, migration, and adhesion.

## 5. Conclusions

Overall, our data identified key pathways associated with the anti-atherosclerotic properties of punicalagin in THP-1 macrophages. Punicalagin’s mechanism of action relies on the regulation of multiple pathways including lipid metabolism, cell inflammation, proliferation, and migration. Whilst future studies are required to support our findings, we identified with high likelihood key regulators of these process that could be targeted by punicalagin.

## Figures and Tables

**Table 1:** Primer sequences used for quantitative-RT PCR.

Gene	Forward Primer	Reverse Primer	Ref.
MSR1	ATTGCCCTTTACCTCCTCGT	TCATTTCCTTTCCCGTGAG	[24]
CD36	AGATGCAGCCTCATTTCCAC	GCCTTGGATGGAAGAACAAA	[24]
NR1H3	AAGCCCTGCATGCCTACGT	TGCAGACGCAGTGCAAACA	[25]
GAPDH	CTTTTGCGTCGCCAGCCGAG	GCCCAATACGACCAAATCCGTTGACT	[26]

**Table 2:** Top 10 significantly upregulated genes.

Gene Symbol	*p*-Value	Fold-Change (Treated vs. Control)
*FABP4*	0.00261999	39.7893
*CD36*	0.000175484	19.5949
*MSR1*	0.000696357	16.8431
*CEMIP*	0.000847506	10.5071
*LPL*	0.000899872	10.0902
*TDO2*	0.0102073	8.73151
*GPC4*	0.0001794	8.39639
*SLC8A1*	0.00194115	7.82049
*SERPINE2*	0.00115135	7.67443
*FCER2*	0.00844659	7.00205

**Table 3:** Top 10 significantly downregulated genes.

Gene Symbol	*p*-Value	Fold-Change (Treated vs. Control)
*MT2A*	0.00236401	−10.6361
*MT1P1*	0.00268895	−4.41548
*FAM207A*	0.0407155	−2.72934
*PLAGL1*	0.00595623	−2.62005
*SOD2*	0.00148297	−2.60906
*MYBPH*	0.00864804	−2.58598
*FKBP5*	0.000695019	−2.4766
*TRAF1*	0.00389735	−2.40964
*NR4A2*	0.0183868	−2.37157
*SNORD1A*	0.0350301	−2.36584

**Table 4:** Top 10 canonical pathways revealed by Ingenuity pathway analysis on 373 genes.

Ingenuity Canonical Pathways	−log (*p*-Value)	z-Score	Molecules
Cholesterol Biosynthesis I	11.3	2.828	CYP51A1, DHCR24, DHCR7, EBP, HSD17B7, LBR, MSMO1, SC5D
Cholesterol Biosynthesis II (via 24,25-dihydrolanosterol)	11.3	2.828	CYP51A1, DHCR24, DHCR7, EBP, HSD17B7, LBR, MSMO1, SC5D
Cholesterol Biosynthesis III (via Desmosterol)	11.3	2.828	CYP51A1, DHCR24, DHCR7, EBP, HSD17B7, LBR, MSMO1, SC5D
Superpathway of Cholesterol Biosynthesis	9.31	3	CYP51A1, DHCR24, DHCR7, EBP, HMGCR, HSD17B7, LBR, MSMO1, SC5D
LXR/RXR Activation	8.97	1.265	ABCG1, AGT, APOC1, APOE, CD36, CYP51A1, HMGCR, LDLR, LPL, LY96, MSR1, NR1H3, SCD, SERPINF1, TLR4
Hepatic Fibrosis/Hepatic Stellate Cell Activation	7.22	0	AGT, COL4A1, COL4A2, COL6A1, EDNRA, FN1, IFNGR1, IGFBP3, LY96, MET, MMP1, PDGFC, TGFB2, TGFB3, TIMP2, TLR4
Inhibition of Matrix Metalloproteases	6.79	0	LRP1, MMP1, MMP12, MMP8, RECK, SDC2, THBS2, TIMP2
Zymosterol Biosynthesis	6.02	2	CYP51A1, HSD17B7, LBR, MSMO1
Phagosome Formation	5.24	0	C3AR1, C5AR1, FCER1G, FCER2, FCGR1A, FN1, ITGA3, ITGA4, MSR1, TLR1, TLR4
Caveolar-mediated Endocytosis Signaling	4.65	0	CAV1, HLA-A, INSR, ITGA3, ITGA4, ITGA6, ITGAL, ITGB5

**Table 5:** Summarized binding affinities of punicalagin.

Receptor Symbol	PDB ID	Binding Affinity w/Punicalagin	Reference Ligand	Binding Affinity w/Ligand	Common H-Bonding
CD36	5LGD	−9.3 (Kcal/mol)	Palmitic acid	−6.8 (Kcal/mol)	0
TLR4	2Z65	−9.0 (Kcal/mol)	Pinocembrin	−8.2(Kcal/mol)	7
MSR1	Modelled	−7.4 (Kcal/mol)	-	−	-
LRP1	1CR8	−6.4 (Kcal/mol)	-	−	-
NR1H3	3IPQ	−7.1 (Kcal/mol)	GW3965	−13.9 (Kcal/mol)	0
TRAF1	5EIT	−9.0 (Kcal/mol)	-	−	-
PPAR-γ	4EMA	−8.7 (Kcal/mol)	Lobeglitazone	−8.6 (Kcal/mol)	3

## Data Availability

Our data has been submitted to GEO with accession number: GSE160430. https://www.ncbi.nlm.nih.gov/geo/query/acc.cgi?acc=GSE160430 (accessed on 15 January 2022).

## Supplementary Materials

The following supporting information can be downloaded at: https://www.mdpi.com/article/10.3390/cimb44050145/s1, Table S1: Differentially expressed genes (DEGs), Included 347 up-regulated and 26 down-regulated with a cut-off of *p* value < 0.05 and fold change more than ±2. Table S2: Canonical pathways generated by ingenuity pathway analysis (IPA). Table S3: Disease functions generated by ingenuity pathway analysis (IPA). Table S4: Top10 predicted z scores of diseases or functions (inhibition or activation).

## References

[1] Cardiovascular Diseases (CVDs). https://www.who.int/news-room/fact-sheets/detail/cardiovascular-diseases-(cvds).

[2] (2013). WHO Global Action Plan for the Prevention and Control of Noncommunicable Diseases 2013–2020.

[3] AJ L. (2000). Atherosclerosis. Nature.

[4] Frostegård J. (2013). Immunity, atherosclerosis and cardiovascular disease. BMC Med..

[5] Frostegard J., Ulfgren A.-K., Nyberg P., Hedin U., Swedenborg J., Andersson U., Hansson G.K. (1999). Cytokine expression in advanced human atherosclerotic plaques: Dominance of pro-inflammatory (Th1) and macrophage-stimulating cytokines. Atherosclerosis.

[6] Cheng Y.-C., Sheen J.-M., Hu W.L., Hung Y.-C. (2017). Polyphenols and Oxidative Stress in Atherosclerosis-Related Ischemic Heart Disease and Stroke. Oxid. Med. Cell. Longev..

[7] Tresserra-Rimbau A., Rimm E.B., Medina-Remón A., Martínez-González M.A., de la Torre R., Corella D., Salas-Salvadó J., Gómez-Gracia E., Lapetra J., Arós F. (2014). Inverse association between habitual polyphenol intake and incidence of cardiovascular events in the PREDIMED study. Nutr. Metab. Cardiovasc. Dis..

[8] Tangney C., Rasmussen H.E. (2013). Polyphenols, Inflammation, and Cardiovascular Disease. Curr. Atheroscler. Rep..

[9] Heber D. (2011). Pomegranate Ellagitannins.

[10] Gil M.I., Tomas-Barberan F.A., Hess-Pierce B., Holcroft D.M., Kader A.A. (2000). Antioxidant activity of pomegranate juice and its relationship with phenolic composition and processing. J. Agric. Food Chem..

[11] Aviram M., Rosenblat M. (2012). Pomegranate Protection against Cardiovascular Diseases. Evid. Based. Complement. Alternat. Med..

[12] Aviram M., Dornfeld L., Rosenblat M., Volkova N., Kaplan M., Coleman R., Hayek T., Presser D., Fuhrman B. (2000). Pomegranate juice consumption reduces oxidative stress, atherogenic modifications to LDL, and platelet aggregation: Studies in humans and in atherosclerotic apolipoprotein E-deficient mice. Am. J. Clin. Nutr..

[13] De Nigris F., Williams-Ignarro S., Lerman L.O., Crimi E., Botti C., Mansueto G., D’Armiento F.P., De Rosa G., Sica V., Ignarro L.J. (2005). Beneficial Effects of Pomegranate Juice on Oxidation-Sensitive Genes and Endothelial Nitric Oxide Synthase Activity at Sites of Perturbed Shear Stress. Proc. Natl. Acad. Sci. USA.

[14] Aviram M., Volkova N., Coleman R., Dreher M., Reddy M.K., Ferreira D., Rosenblat M. (2008). Pomegranate phenolics from the peels, arils, and flowers are antiatherogenic: Studies in vivo in atherosclerotic apolipoprotein E-deficient (E0) mice and in vitro in cultured macrophages and lipoproteins. Proc. J. Agric. Food Chem..

[15] Atrahimovich D., Samson A.O., Khattib A., Vaya J., Khatib S. (2018). Punicalagin Decreases Serum Glucose Levels and Increases PON1 Activity and HDL Anti-Inflammatory Values in Balb/c Mice Fed a High-Fat Diet. Oxid. Med. Cell. Longev..

[16] De Nigris F., Williams-Ignarro S., Sica V., Lerman L.O., D’Armiento F.P., Byrns R.E., Casamassimi A., Carpentiero D., Schiano C., Sumi D. (2007). Effects of a Pomegranate Fruit Extract rich in punicalagin on oxidation-sensitive genes and eNOS activity at sites of perturbed shear stress and atherogenesis. Cardiovasc. Res..

[17] Pomegranate Juice Flavonoids Inhibit Low-Density Lipoprotein Oxidation and Cardiovascular Diseases: Studies in Atherosclerotic Mice and in Humans—PubMed. https://pubmed.ncbi.nlm.nih.gov/12224378/.

[18] Atrahimovich D., Khatib S., Sela S., Vaya J., Samson A.O. (2016). Punicalagin Induces Serum Low-Density Lipoprotein Influx to Macrophages. Oxid. Med. Cell. Longev..

[19] Seeram N.P., Henning S.M., Zhang Y., Suchard M., Li Z., Heber D. (2006). Pomegranate juice ellagitannin metabolites are present in human plasma and some persist in urine for up to 48 hours. J. Nutr..

[20] Cao Y., Chen J., Ren G., Zhang Y., Tan X., Yang L. (2019). Punicalagin prevents inflammation in lps-induced raw264.7 macrophages by inhibiting foxo3a/autophagy signaling pathway. Nutrients.

[21] BenSaad L.A., Kim K.H., Quah C.C., Kim W.R., Shahimi M. (2017). Anti-inflammatory potential of ellagic acid, gallic acid and punicalagin A&B isolated from Punica granatum. BMC Complement. Altern. Med..

[22] Michael D.R., Salter R.C., Ramji D.P. (2012). TGF-β inhibits the uptake of modified low density lipoprotein by human macrophages through a Smad-dependent pathway: A dominant role for Smad-2. Biochim. Biophys. Acta-Mol. Basis Dis..

[23] Almowallad S., Huwait E., Al-Massabi R., Saddeek S., Gauthaman K., Prola A. (2020). Punicalagin regulates key processes associated with atherosclerosis in thp-1 cellular model. Pharmaceuticals.

[24] Xu W., Yu L., Zhou W., Luo M. (2006). Resistin increases lipid accumulation and CD36 expression in human macrophages. Biochem. Biophys. Res. Commun..

[25] Saenz J., Santa-María C., Reyes-Quiroz M.E., Geniz I., Jiménez J., Sobrino F., Alba G. (2018). Grapefruit Flavonoid Naringenin Regulates the Expression of LXRα in THP-1 Macrophages by Modulating AMP-Activated Protein Kinase. Mol. Pharm..

[26] Gallagher H., Williams J.O., Ferekidis N., Ismail A., Chan Y.H., Michael D.R., Guschina I.A., Tyrrell V.J., O’Donnell V.B., Harwood J.L. (2019). Dihomo-γ-linolenic acid inhibits several key cellular processes associated with atherosclerosis. Biochim. Biophys. Acta-Mol. Basis Dis..

[27] Pepino M.Y., Kuda O., Samovski D., Abumrad N.A. (2014). Structure-function of CD36 and importance of fatty acid signal transduction in fat metabolism. Annu. Rev. Nutr..

[28] Pan L., Duan Y., Ma F., Lou L. (2020). Punicalagin inhibits the viability, migration, invasion, and EMT by regulating GOLPH3 in breast cancer cells. J. Recept. Signal Transduct..

[29] Tang J.M., Min J., Li B.S., Hong S.S., Liu C., Hu M., Li Y., Yang J., Hong L. (2016). Therapeutic Effects of Punicalagin Against Ovarian Carcinoma Cells in Association with β-Catenin Signaling Inhibition. Int. J. Gynecol. Cancer.

[30] Malik A., Mukhtar H. (2006). Prostate cancer prevention through pomegranate fruit. Cell Cycle.

[31] Zhao S.J., Kong F.Q., Jie J., Li Q., Liu H., Di Xu A., Yang Y.Q., Jiang B., Wang D.D., Zhou Z.Q. (2020). Macrophage MSR1 promotes BMSC osteogenic differentiation and M2-like polarization by activating PI3K/AKT/GSK3β/β-catenin pathway. Theranostics.

[32] Alvarez-Cubero M.J., Saiz M., Martinez-Gonzalez L.J., Alvarez J.C., Lorente J.A., Cozar J.M. (2013). Genetic analysis of the principal genes related to prostate cancer: A review. Urol. Oncol. Semin. Orig. Investig..

[33] Quirós-Fernández R., López-Plaza B., Bermejo L.M., Palma-Milla S., Gómez-Candela C. (2019). Supplementation with Hydroxytyrosol and Punicalagin Improves Early Atherosclerosis Markers Involved in the Asymptomatic Phase of Atherosclerosis in the Adult Population: A Randomized, Placebo-Controlled, Crossover Trial. Nutrients.

[34] Metwally H., Tanaka T., Li S., Parajuli G., Kang S., Hanieh H., Hashimoto S., Chalise J.P., Gemechu Y., Standley D.M. (2020). Noncanonical STAT1 Phosphorylation Expands Its Transcriptional Activity into Promoting LPS-Induced IL-6 and IL-12p40 Production. Sci. Signal..

[35] Lalani A.I., Zhu S., Gokhale S., Jin J., Xie P. (2018). TRAF Molecules in Inflammation and Inflammatory Diseases. Curr. Pharmacol. Rep..

[36] Mazumder M.K., Choudhury S., Borah A. (2019). An in silico investigation on the inhibitory potential of the constituents of Pomegranate juice on antioxidant defense mechanism: Relevance to neurodegenerative diseases. IBRO Rep..

